# What is in the feedback? Effect of induced happiness vs. sadness on probabilistic learning with vs. without exploration

**DOI:** 10.3389/fnhum.2015.00584

**Published:** 2015-10-29

**Authors:** Jasmina Bakic, Rudi De Raedt, Marieke Jepma, Gilles Pourtois

**Affiliations:** ^1^Department of Experimental Clinical and Health Psychology, Ghent UniversityGhent, Belgium; ^2^Department of Psychology and Neuroscience, University of Colorado BoulderBoulder, CO, USA

**Keywords:** reinforcement learning, mood, exploration-exploitation, emotion, probabilistic learning

## Abstract

According to dominant neuropsychological theories of affect, emotions signal salience of events and in turn facilitate a wide spectrum of response options or action tendencies. Valence of an emotional experience is pivotal here, as it alters reward and punishment processing, as well as the balance between safety and risk taking, which can be translated into changes in the exploration-exploitation trade-off during reinforcement learning (RL). To test this idea, we compared the behavioral performance of three groups of participants that all completed a variant of a standard probabilistic learning task, but who differed regarding which mood state was actually induced and maintained (happy, sad or neutral). To foster a change from an exploration to an exploitation-based mode, we removed feedback information once learning was reliably established. Although changes in mood were successful, learning performance was balanced between the three groups. Critically, when focusing on exploitation-driven learning only, they did not differ either. Moreover, mood valence did not alter the learning rate or exploration *per se*, when titrated using complementing computational modeling. By comparing systematically these results to our previous study (Bakic et al., 2014), we found that arousal levels did differ between studies, which might account for limited modulatory effects of (positive) mood on RL in the present case. These results challenge the assumption that mood valence alone is enough to create strong shifts in the way exploitation or exploration is eventually carried out during (probabilistic) learning. In this context, we discuss the possibility that both valence and arousal are actually necessary components of the emotional mood state to yield changes in the use and exploration of incentives cues during RL.

## Introduction

Many students would agree that studying for an exam after a heartbreak is a particularly hard thing to do. On the other hand, some professors would argue that, if one wants to excel, also the happy, falling in love phase is best avoided altogether. Even if it was possible, would it really be best if emotions were somehow hushed and kept at bay in order to learn? Or is it possible that both happiness and sadness can enhance learning? If so, which one works better?

Emotions are complex, multi-faceted phenomena that signal importance of events and guide actions to maximize benefits and minimize damage. From an evolutionary perspective, development of such variety and richness of emotions as we know today enabled more flexible, more adaptive functioning, and ultimately, a wider spectrum of response options (Lang and Bradley, 2010). In that sense, valence of emotional experiences plays a pivotal role: positive emotions (such as happiness, joy, amusement, pleasantness) are hypothesized to signal safety and instigate creativity, exploration, playfulness, and risk-taking. In contrast, negatively valenced emotions, such as fear, sadness, anger, disgust, or frustration, signal threat and the need to recruit additional resources to deal with potential harm or loss (Isen, 1993; Ashby et al., 1999; Fredrickson, 2004).

From this initial premise, different expectations about the effects of positive and negative emotions on cognition and behavior can be derived. Most research on the topic was performed by inducing mood, using different strategies (including movie clips, images, music, autobiographical pieces, guided imagery; see Martin, 1990; Westermann et al., 1996). Moods are considered to be more enduring and milder than emotions, and are not directed towards a certain entity, but are rather “non-focal” (Bolte and Goschke, 2010). Mood effects have been examined in the area of creative thinking (Isen, 1984; Isen et al., 1985, 1987), attention (Huntsinger, 2012; Vanlessen et al., 2014), and cognitive control (van Steenbergen et al., 2010; Fröber and Dreisbach, 2014). Effects of mood on performance are rather mixed though, with some studies showing that positive mood does not necessarily translate into improved (behavioral) performance (van Steenbergen et al., 2010; Braem et al., 2012; Zwosta et al., 2013), or that negative mood automatically leads to detrimental effects for cognition and behavior (for example, see Cavanagh et al., 2011). Hence, the prevailing notion that positive emotions are unequivocally beneficial for functioning, while negative ones are necessarily detrimental has been challenged recently. For example, recent studies showed that positive affect can actually lower proactive control (Dreisbach, 2006; Vanlessen et al., 2015), which, depending on the task at hand, can be either detrimental or beneficial. Moreover, positive affect can foster the dominant cognitive style, while negative affect can counteract it, an observation that speaks against the idea that positive valence is unconditionally related to a broad focus and enhanced flexibility, while negative valence is related to a narrow focus and enhanced rigidity (Hunsinger et al., 2012). In this context, positive mood does not simply correspond to the mere opposite of negative mood along a valence dimension or continuum.

As a matter of fact, reinforcement learning (RL) is a particularly good candidate as a process to be modulated by mood, because by definition, it relies directly on the processing of positive vs. negative information or incentives to achieve a goal at hand. Stimulus-response associations (S-R) are being formed in a trial-and-error fashion, based on externally provided feedback, reward or punishment, about one’s own actions (Sutton and Barto, 1998). If current mood provides an emotional context for the learning situation, then it could change the salience of error and reward, or how threatening and appetitive they are eventually perceived, and in turn processed. A performance monitoring system in charge of learning will value opportunities and threats in surroundings differently depending on the current state and the needs of the organism. Most theories of RL argue that performance optimization is based on the right amount of exploitation of rewarding options, and exploration of less known, but potentially even more beneficial alternatives (Aston-Jones and Cohen, 2005; Behrens et al., 2007; Cohen et al., 2007; Jepma and Nieuwenhuis, 2011). These two concurrent processes, exploration and exploitation, have complementary benefits or functions: while it is important to keep current goals in mind and not allow for distractions (i.e., favor exploitation), it is at the same time important to keep the environment in check for potential changes that might reliably influence performance (i.e., foster exploration). We hypothesize that this trade-off between exploration and exploitation might be susceptible to changes in the current mood state of the participant. If positive mood leads to more exploration of less known options, while sad mood is accompanied by a more stringent focus (Bolte and Goschke, 2010), oriented towards negative information, then this effect should be visible in the exploration-exploitation trade-off during RL. Moreover, we can expect that mood manipulation will also influence the usage of positive and negative feedback for response updating, such that happy participants could update more based on positive (than negative) feedback, while sad subjects could avoid negative feedback.

Along these lines, Unger et al. (2012) have shown that, in a learning paradigm, inducing a feeling of performance-related failure changes the strategy towards more error-driven behavioral control, while it concurrently increases early electrophysiological markers of error monitoring (at the level of the error related negativity, ERN). In a previous study, using a probabilistic learning paradigm, we also showed that happy mood increased the ERN when learning was deterministic, and was associated with an augmented learning rate (but not exploration, see Bakic et al., 2014). More specifically, we used a probabilistic learning task (Eppinger et al., 2008) in which the different S-R associations that had to be learned across multiple and successive encounters had actually different reward probabilities, unknown to the participants. This situation usually creates a certain amount of uncertainty that learning agents have to overcome in order to optimize their learning performance. Presumably, this uncertainty might be dealt with differently depending on the current emotional state of the participant. Even though in this study we demonstrated that, by modulating the current mood of the participant, we were able to modulate the learning rate (accompanied by change on the electrophysiological level as well, more precisely for the ERN component; see Bakic et al., 2014), we failed however to show that positive mood led to clear benefits or impairments in the actual learning performance during this probabilistic task (i.e., happy participants did not perform better or worse than neutral participants during RL).

Accordingly, in the present study, we sought to adapt this experimental paradigm (see Bakic et al., 2014) in a way that would allow us to maximize the chance to capture such a difference at the behavioral level between the two groups. For this purpose, in addition to a standard initial learning phase (consisting of trials made each time of S-R-feedback associations) that is identical as in previous research (Eppinger et al., 2008; Bakic et al., 2014), we added a second phase, where feedback on task performance was omitted. In other words, during this second phase (when learning was already established), we changed the trial structure in such a way that a S-R-feedback sequence was changed to S-R one, preventing participants from using feedback information (and thereby exploration) to guide learning. At this point, participants could only use stored value estimates, and were no longer able to track state transitions of value. Based on the results obtained in our previous study, we already knew that S-R associations were already formed during the first phase before they moved to the second one. More specifically, internalization of task rules took place and externally provided feedback was no longer necessary to perform the task accurately. This is consistent with the assumption that exploration of different response alternatives was no longer needed, and the (direct) exploitation of the acquired knowledge could be carried out. Using this specific manipulation, we wanted to examine whether creating such clear-cut difference between the exploration and exploitation stage of the task could eventually lead to a clearer difference at the behavioral level between the two groups than in our previous study (Bakic et al., 2014). Additionally, other than comparing only happy and neutral mood, in the current study, we added a third group of participants who received a similar mood induction procedure (MIP) but with a sad content. This way, we could assess whether sadness might perhaps produce different effects on RL compared to happiness, thereby confirming that mood valence plays a critical role in triggering specific changes during RL.

To summarize, the goal of this study was to test the effects of inducing happy, neutral or sad mood on RL (operationalized using a probabilistic learning task; see Bakic et al., 2014), when this process was broken down into two consecutive phases: an initial learning phase relying on the use of external feedback information to guide learning (where both exploration and exploitation are used in synergy), followed by a second phase where feedback was omitted (and exploitation alone is encouraged). Our experimental design involved comparisons of three groups of participants differing from one another regarding the actual mood state induced (happy, neutral, or sad), but using the same guided imagery procedure (Holmes and Mathews, 2010). Based on our first study (Bakic et al., 2014), during the first part of the task, we did not expect to find group differences in rough measures of learning (e.g., accuracy). We surmised, however, that the happy group could show a higher learning rate (with no change in exploration), compared to the neutral (and/or sad) group. If sad mood influences learning performance in an opposite manner compared to the positive mood group, then we could expect a lower learning rate in this group compared to the two other ones (happy and neutral). Additionally, we predicted that during the second phase of the task where feedback information was no longer available, happy mood could be associated with a better learning performance than either neutral or sad mood given that this specific mood state could bolster internalization of the task rules and in turn exploitation (Nadler et al., 2010). Alternatively, if positive mood truly fostered the dominant response tendency or cognitive style (Hunsinger et al., 2012), then we could expect that following its induction an increased use of the learned S-R associations could be observed during this part of the experiment where exploitation of prior knowledge was encouraged.

## Materials and Methods

### Participants

Fifty two participants (undergraduate psychology students) took part in the study in exchange for course credits. They were randomly assigned to one of the three mood groups: happy, neutral or sad mood. They were all right-handed, with no past or current neurological or psychiatric problems, they had normal or corrected-to normal vision, and all gave written informed consent prior to the start of the experiment. The data of seven participants were removed according to the following exclusion criteria (see also Bakic et al., 2014). First, one participant was excluded in the happy group because of the lack of a marked increase in happy mood following the MIP compared to the baseline (i.e., the average increase was not different than the baseline value). Likewise, one participant was excluded from the sad group due to the lack of a marked increase in sadness relative to the baseline mood measurement. Finally, two participants were excluded from the neutral mood group because their average happiness level was higher than the mean of the happy group, whereas no change in mood was expected to take place in this control group. Second, participants showing no learning during the main task (i.e., their learning curves did not differ from chance level) were excluded as well (*n* = 3; one in each group). Note that the behavioral results obtained for the accuracy, RT and learning rate data remained unchanged when including them in the statistical analyses. However, because they did not show learning, their data were deemed noisy and they were therefore removed from the subsequent statistical analyses. The final sample consisted of 45 participants (mean age = 20.62 years, SD = 2.29, 29 females), 14 in the happy, 14 in the neutral, and 17 in the sad mood group. The study was approved by the local ethics committee.

### Mood Induction

We used a previously validated MIP (Vanlessen et al., 2013, 2014; Bakic et al., 2014). Mood was induced by means of a guided imagery procedure, where participants were instructed to vividly imagine reliving either a happy, neutral, or sad (depending on the group they were assigned to) autobiographical memory (Holmes et al., 2006, 2008). First, the participants were trained in taking a field perspective (i.e., imagining from one’s own perspective) during mental imagery. Then they had to choose an appropriate happy/neutral/sad event, an episodic memory that happened at least a week before, and to report explicitly about it. For the recall that would ensue, they were instructed to keep their eyes closed and visualize all the specificities of the memory, and to use the field perspective (Watkins and Moberly, 2009; based on Holmes et al., 2008). The actual recall session was divided into two parts of 30 s each, and in between participants were asked questions about different aspects of the happy/neutral/sad memory they were imagining. Participants were blind to the real purpose of the procedure, believing that it was about remembering an event from the past as vividly as possible (and not about re-experiencing the actual emotion of this specific event). After each mood induction, participants marked on 10 cm horizontal visual analog scales (VAS) their current level of happiness, pleasantness, and sadness, with “neutral” on one end/anchor to “as happy/pleasant/sad as I can imagine” on the other. Arousal was measured on a 9-point Likert scale.

### Probabilistic Learning Task

**Figure 1:**
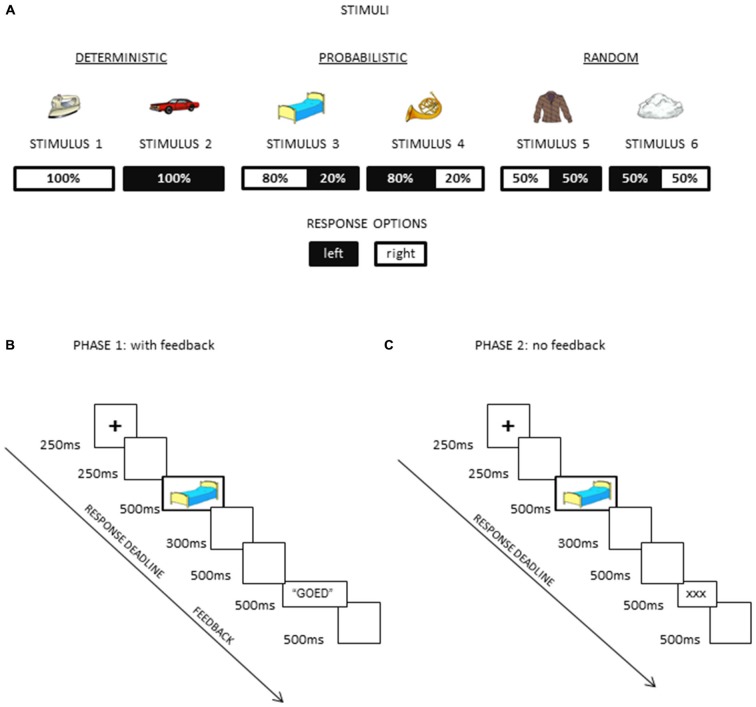
**Schematic illustrations of (A) the task structure with different probabilities assigned to different stimuli, (B) the trial structure in the first phase of the experiment where the feedback was provided after each response (and both exploration and exploitation were therefore required), and (C) the trial structure in the second phase of the experiment where an uninformative feedback was provided (and exploitation was encouraged)**.

A modified version of the probabilistic learning task previously validated by Eppinger et al. (2008) was used in this study, with the first phase of the experiment being the same as in previous studies (Eppinger et al., 2008; Bakic et al., 2014; see Figure 1B) During this phase, participants were asked to decipher and learn, by trial and error, several hidden S-R mappings. For each trial, participants were asked to decide, with a time limit, whether the stimulus shown on the screen was associated with response 1 or 2. Visual feedback regarding the actual choice made by the participant was given following each and every response made during this first phase. Upon completion of this first phase, participants move to the second phase of the experiment, where a generic and uninformative feedback was now presented but task instructions remained unchanged (Figure 1C).

Participants were presented with 6 visual stimuli (A-B-C-D-E-F), belonging to three conditions (unknown to the participants) that differed regarding the actual probability of the S-R mapping (100, 80 or 50%). In the condition 100%, each stimulus of the pair was always associated with one of the two response keys, corresponding to a “deterministic” S-R mapping (i.e., response 1 was always correct for stimulus A, and response 2 for the stimulus B). In the condition 80%, the S-R mapping was “probabilistic”, given that stimulus C was associated 80% of the time with response 1 (and thus 20% of the time with the concurrent response 2), while stimulus D had a symmetric probability for the S-R mapping. Finally, in the condition 50% (“random” S-R mapping), each stimulus of the pair was associated equally often to each of the two response keys (i.e., stimuli E and F were associated 50% of the time with response 1 and 50% of the time with response 2). The structure of the task is presented in Figure 1.

Colorful line drawings (Rossion and Pourtois, 2004) were used as visual stimuli (Figure 1A), presented against a white homogenous background on a 17-inch computer screen. These stimuli were visual objects belonging to different semantic categories (artifacts, buildings, musical instruments, clothes, vehicles, furniture). Their mean size was 7 cm width × 5 cm height, corresponding to 5 × 3, 6 degrees of visual angle at 80 cm viewing distance.

For the first phase of the experiment (Figure 1B), the trial structure was as follows: it began with a fixation cross of 250 ms duration, followed by a 250 ms blank screen. Then, the stimulus was presented for 500 ms, followed by a blank screen lasting 300 ms. Response deadline was set to 800 ms following stimulus onset. After 500 ms, performance feedback was presented for 500 ms. The feedback was provided in the form of a written word (in Dutch) shown in black against a white homogenous background. This word was “goed” (correct), “fout” (incorrect), or “te traag” (too late). The inter-trial interval was constant (500 ms) and it corresponded to a blank screen, after which a new trial ensued. Manual responses (i.e., key presses) were recorded using the Cedrus response box. After participants completed 240 trials (6 stimuli × 40 repetitions), trial structure changed. During this second phase (Figure 1C), trial structure was the same as for the first phase of the experiment, with the following notable exception: instead of a meaningful feedback response (informing the participant about his actual accuracy) appearing on the screen after each manual response, there were three “×” signs shown as visual feedback each time (in the same location and for the same duration as in the previous phase of the experiment). In this way, trial structure remained identical, the only difference being the lack of informative external feedback. Participants performed 120 trials (6 stimuli × 20 repetitions) with this uninformative feedback (“no feedback” condition hereafter).

Each participant completed two blocks of 360 trials (240 with and 120 without feedback). Each block had six different stimuli. Accordingly, participants had to learn six new S-R associations in each block. Trial order within a block as well as the order of the two blocks were alternated across participants.

### Procedure

**Figure 2:**
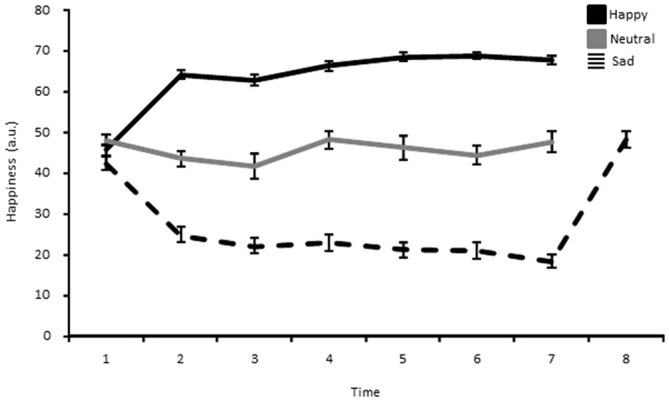
**Happiness ratings shown separately for the happy, neutral and sad mood group as a function of time.** Each point represents the mean and the error bar corresponds to 1 standard error of the mean.

In order to get acquainted with the task, participants first completed a short practice session of 20 trials. Next, a happy, neutral, or sad mood was induced by means of the MIP before the beginning of the first block. In order to sustain the targeted mood throughout the whole experimental session, the same MIP was briefly rehearsed (5 min) during both blocks, every 120 trials (corresponding to two bins; see data analysis here below). The same procedure was also repeated during the break between two blocks. Hence, in total, participants encountered the MIP 7 times. Additionally, participants assigned to the sad group received one more MIP at the very end of the experiment. This MIP consisted of actively reliving a happy past memory episode (very much like what was made in the happy mood group) in order to make sure that these participants (sad mood group) would not leave the lab with a lingering sad mood. Self-ratings after this MIP showed that happiness ratings for the sad mood group went back to a neutral mood baseline, being in turn comparable to those of the neutral mood group after the experiment (see Figure 2).

In order to strengthen the effect of mood, an evaluative feedback was added (rewarding in the happy mood group, neutral in the neutral group, and mildly negative in the sad mood group) at the end of each block. This (bogus) feedback consisted of a small text fragment shown on the screen, informing participants that they had to wait briefly until the computer had calculated online their learning performance up to that trial number. After a few seconds, an Excel-like scatter plot appeared on the screen, showing them their performance level allegedly relative to a group of peers. Their score was indicated by means of a color dot. This dot was positioned systematically either higher up in the distribution of scores for participants in the happy mood group, somewhere in the middle of the distribution for those belonging to the neutral mood group, and slightly lower for the participants in the sad mood group. Next to this scatter plot, a specific written message was included. It informed them to try to keep the same level of performance or perform better if possible. Manipulation checks based on VASes (see “Results” Section below) confirmed that this procedure (combined with the MIP) actually produced the desired effects: an increase of happiness in the happy mood group, with no change in affect (neither happy, nor sad) in the neutral mood group, and a decrease of happiness in the sad mood group. However, it should be mentioned that because we used the MIP in conjunction with this bogus feedback manipulation, the changes in mood observed (at the subjective level; see results here below) were likely to be explained no only by the MIP, but also (albeit to a lower degree) by some motivational processes involved in the processing of this (bogus) feedback information. However, we have good reasons to believe that the change in happiness (or sadness in the sad mood group) was mainly due to the MIP and the use of guided imagery (see also Vanlessen et al., 2013, 2014), and not so much to this (infrequent) feedback manipulation that occurred only four times during the course of the experiment. Moreover, after each block, participants were asked to indicate, for each of the 6 stimuli, the clarity and certainty of each of the six S-R associations, by means of a horizontal 10 cm VAS. Furthermore, they were asked to rate the amount of positive vs. negative feedback they thought they received during this last block (using a 10 cm VAS going from “exclusively negative” to “exclusively positive”), as well as how much they liked or disliked these positive vs. negative feedback when receiving them (using a Likert scale spanning from 0 to 100).

Finally, participants were asked to fill out two trait-related questionnaires: the Beck Depression Inventory (Beck et al., 1996), and the Resilience scale translated in Dutch (Portzky et al., 2010). The whole experiment lasted for about 2 h.

### Data Analysis

#### Mood Manipulation

The efficiency of the increase/decrease in happy mood (relative to the neutral group) following the MIP was assessed by means of mixed model ANOVAs with group (*n* = 3) as between subjects factor and time (*n* = 7) as within subjects factor.

#### Accuracy Analyses

Accuracy data were expressed in proportions of correct responses from the total number of trials, separately for each probability (*n* = 3). Moreover, for each probability separately (2 stimuli × 40 repetitions), changes of learning performance as a function of time were captured by grouping the data into bins of equal sizes (i.e., 20 trials; see Eppinger et al., 2008; Bakic et al., 2014 for a similar approach). These data were then submitted to a mixed model ANOVA with Group (*n* = 3) as between subjects factor, and Probability (*n* = 3) and Bin (*n* = 4) as within subjects factors. Additionally, in order to compare possible differences between learning with vs. without feedback, we averaged the scores of bins 3 and 4 together (exploitation and exploration), and bin 5 and 6 (exploitation only), and submitted these mean values to a mixed model ANOVA with Group as between subjects factor, and Probability and Phase as within subject factors. Where necessary, Greenhouse-Geisser correction for sphericity was performed, and corrected *p* values were reported, together with uncorrected eta square measure of effect size.

#### RL Model

For the first phase of the experiment (with feedback information provided to the participants), we used two complementary measures based on the modeling procedure described previously in Bakic et al. (2014). We computed first the learning rate parameter (α), which determines the impact of the most recent feedback on the current S-R association, such that higher learning rates correspond to larger fluctuations in response behavior from trial to trial while lower learning rates index more stable response behavior. We also calculated a second parameter, β, which is a “noise” parameter that reflects how random choices are (and hence it provides an indirect measure of exploration).

## Results

### Mood

The analysis of the MIP ratings showed a significant Time*Group interaction for pleasantness (*F*_(7.25,152.22)_ = 8.53, *p* < 0.01, η^2^ = 0.29), happiness (*F*_(9.45,198.40)_ = 11.18, *p* < 0.01, η^2^ = 0.35), sadness (*F*_(9.84,81.19)_ = 6.98, *p* < 0.01, η^2^ = 0.25), and arousal (*F*_(8.22,172.53)_ = 2.36, *p* < 0.05, η^2^ = 0.10). The main effect of Group was also significant for pleasantness (*F*_(2,42)_ = 42.02, *p* < 0.01, η^2^ = 0.67), happiness (*F*_(2,42)_ = 43.71, *p* < 0.01, η^2^ = 0.68), sadness (*F*_(2,42)_ = 24.71, *p* < 0.01, η^2^ = 0.54), and arousal (*F*_(2,42)_ = 5.79, *p* < 0.05, η^2^ = 0.22). Independent samples *t*-tests for direct comparisons between the happy and the neutral, and the neutral and the sad group showed that there were no significant differences at baseline, whereas in each subsequent measure (hence following the MIP each time) the happy group showed an increase compared to the neutral group, while the sad group showed a marked decrease in levels of pleasantness (Table 1). The same was true for happiness ratings (Figure 2; Table 2). Independent *t*-tests for the sadness ratings (Table 3) showed that the happy and the neutral group had comparable, low and unchanged levels of sadness, whereas in the sad group sadness increased after the first MIP and stayed significantly higher than in the neutral group throughout the duration of the experiment (except for the last measurement following a positive MIP meant to restore a neutral mood state in this group; see methods). More specifically, a paired sample *t*-test for the sad group showed that after the final happy MIP (*M* = 14.5, SD = 12.2), this group had significantly lower sadness scores than after the last sad MIP (*M* = 31.36, SD = 17.64), (*t*_(16)_ = 4.51, *p* < 0.01). At the same time, happiness scores increased significantly from last sad MIP (*M* = 18.43, SD = 14.14) to the happy MIP (*M* = 48.43, SD = 17.09), (*t*_(16)_ = −7.04, *p* < 0.01). The happy and the sad group did not differ significantly in arousal levels (Table 4), but the sad group showed somewhat lower arousal scores than the neutral group.

**Table 1:** **Results of the Pleasantness scores**.

Measure point	Pleasantness				
				*t*-test	
	Happy	Neutral	Sad	Happy vs. Neutral	Neutral vs. Sad
Baseline	47.32 (15.91)	46.38 (15.93)	43.90 (13.28)	0.16	0.47
1	65.01 (7.14)	47.86 (12.49)	32.90 (12.23)	4.46**	3.36**
2	64.57 (10.18)	45.25 (21.56)	24.67 (11.96)	3.03**	3.36**
3	66.36 (8.38)	51.14 (13.97)	27.68 (14.72)	3.49**	4.52**
4	67.12 (10.78)	49.06 (19.19)	22.47 (15.30)	3.07**	4.30**
5	68.35 (6.80)	45.74 (20.24)	24.39 (15.08)	3.96**	3.36**
6	67.46 (7.71)	46.62 (21.47)	21.60 (13.15)	3.42**	4.00**

Means (+1 Standard Deviation) and results of the group comparison (based on independent-samples t-tests) between the Happy and Neutral (df = 26) or the Neutral and Sad (df = 29) mood Group. *p < 0.05, **p < 0.01.

**Table 2:** **Results of the Happiness scores**.

Measure point	Happiness				
				*t*-test	
	Happy	Neutral	Sad	Happy vs. Neutral	Neutral vs. Sad
Baseline	45.59 (10.84)	48.05 (10.04)	42.55 (15.55)	−0.62	1.14
1	64.16 (9.19)	43.64 (13.33)	24.75 (15.03)	4.74**	3.66**
2	62.87 (9.79)	41.73 (22.46)	22.12 (15.69)	3.23**	2.87**
3	66.28 (8.26)	48.29 (16.76)	23.05 (15.38)	3.60**	4.37**
4	68.56 (7.88)	46.27 (20.40)	21.30 (16.61)	3.81**	3.86**
5	68.81 (5.97)	44.28 (17.64)	21.07 (16.91)	4.93**	3.73**
6	67.88 (7.52)	47.71 (20.22)	18.43 (14.14)	3.50**	4.74**

Means (+1 Standard Deviation) and results of the group comparison (based on independent-samples *t*-tests) between the Happy and Neutral (df = 26) and the Neutral and Sad (df = 29) mood Group. *p < 0.05, **p < 0.01.

**Table 3:** **Results of the Sadness scores**.

Measure point	Sadness				
				*t*-test	
	Happy	Neutral	Sad	Happy vs. Neutral	Neutral vs. Sad
Baseline	13.59 (10.48)	8.82 (8.38)	11.64 (9.47)	1.33	−0.87
1	6.39 (7.54)	7.04 (5.80)	31.67 (14.68)	−0.25	−5.90**
2	5.41 (7.32)	9.32 (12.17)	33.39 (15.73)	−1.03	−4.68**
3	7.21 (7.95)	8.06 (6.70)	33.51 (17.83)	−0.31	−5.04**
4	6.28 (7.25)	5.66 (6.98)	28.06 (18.06)	0.23	−4.37**
5	5.31 (6.61)	9.35 (11.25)	27.60 (19.17)	−1.16	−3.14**
6	8.82 (10.02)	7.94 (6.32)	31.36 (17.64)	0.28	−4.72**

Means (+1 Standard Deviation) and results of the group comparison (based on independent-samples t-tests) between the Happy and Neutral (df = 26) or the Neutral and Sad (df = 29) mood Group. *p < 0.05, **p < 0.01.

**Table 4:** **Results of the Arousal scores**.

Measure point	Arousal				
				*t*-test	
	Happy	Neutral	Sad	Happy vs. Neutral	Neutral vs. Sad
Baseline	4.79 (1.12)	5.07 (1.82)	3.65 (1.46)	−0.50	2.43*
1	5.93 (1.77)	5.21 (1.37)	4.29 (1.86)	1.19	1.54
2	5.93 (2.09)	5.64 (1.69)	3.47 (1.59)	0.40	3.68**
3	5.64 (2.41)	5.21 (1.37)	3.76 (1.48)	0.58	2.81*
4	5.71 (2.64)	4.64 (1.60)	4.12 (1.65)	1.30	0.89
5	5.93 (2.20)	4.93 (1.69)	4.18 (1.74)	1.35	1.21
6	5.86 (2.31)	3.86 (1.46)	3.94 (1.64)	2.73*	−0.15

Means (+1 Standard Deviation) and results of the group comparison (based on independent-samples t-tests) between the Happy and Neutral (df = 26) or the Neutral and Sad (df = 29) mood Group. *p < 0.05, **p < 0.01.

### Too Late Responses

The number of too late responses was modest (*M* = 1.64, SD = 0.93) and not different between the three mood groups (*p*’s > 0.05). There was a significant Group*Bin interaction (*F*_(10,210)_ = 3.02, *p* < 0.01, η^2^ = 0.13), showing that neutral group had a larger number of too late responses compared to the other two groups for the final two bins without feedback. Additionally, there was a significant main effect of Probability (*F*_(2,84)_ = 9.64, *p* < 0.01, η^2^ = 0.19), and Bin (*F*_(3.71,155.95)_ = 2.94, *p* < 0.05, η^2^ = 0.10). In the deterministic condition (*M* = 1.37, SD = 0.93), the number of too late responses was lower than in the random condition (*M* = 2.02, SD = 1.19), (*t*_(44)_ = −5.20, *p* < 0.01), but not different than in the probabilistic condition (*M* = 1.54, SD = 1.06), (*p* > 0.05). This latter condition differed significantly from the random condition (*t*_(44)_ = −3.48, *p* < 0.01). Moreover, paired samples *t*-tests showed that the number of too late responses differed only between bin1 (*M* = 2.08, SD = 1.39) and bin2 (*M* = 1.76, SD = 1.38) (*t*_(44)_ = 2.34, *p* < 0.05), while the other comparisons between bins did not reach significance (all *p*s > 0.05).

### Accuracy

**Figure 3:**
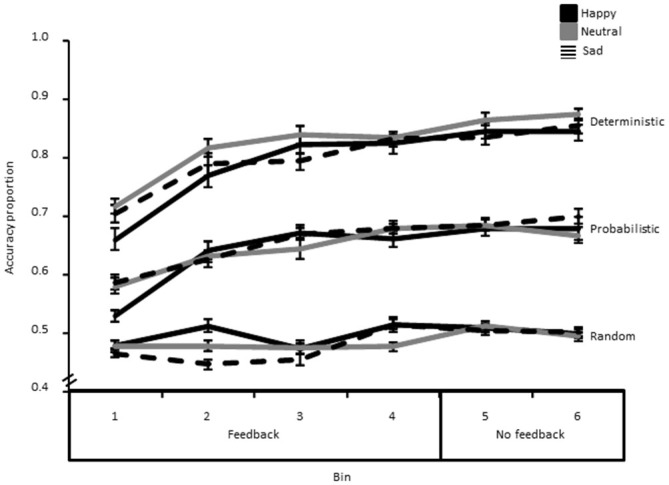
**Accuracy data (i.e., proportion of correct responses) decomposed as a function of bin, probability and group.** The error bar corresponds to one standard error of the mean.

Results showed a significant Probability*Bin interaction (*F*_(10,420)_ = 6.13, *p* < 0.01, η^2^ = 0.13), as well as significant main effects of Probability (*F*_(1.70,71.89)_ = 334.96, *p* < 0.01, η^2^ = 0.89), and Bin (*F*_(3.92,164.70)_ = 34.27, *p* < 0.01, η^2^ = 0.45). This interaction indicated, as can be seen from Figure 3, that accuracy was higher and that learning was steeper in the deterministic than in the probabilistic condition, while there was no learning (across time) whatsoever in the random condition.

Next, we averaged performance for the two last Bins in the learning phase with feedback and compared it to the two bins of the “no feedback” phase in order to assess whether learning still increased once feedback information on task performance was removed, and exploitation only was required (Figure 3). This analysis showed a significant main effect of Phase (*F*_(1,42)_ = 10.02, *p* < 0.01, η^2^ = 0.19), suggesting that learning still increased reliably after removing the feedback. There was also a significant main effect of Probability (*F*_(2,84)_ = 365.28, *p* < 0.01, η^2^ = 0.90). However, there were no significant group-related effects (all *p*^′^s > 0.05).

### Reaction Times (RTs) for Correct Responses

**Figure 4:**
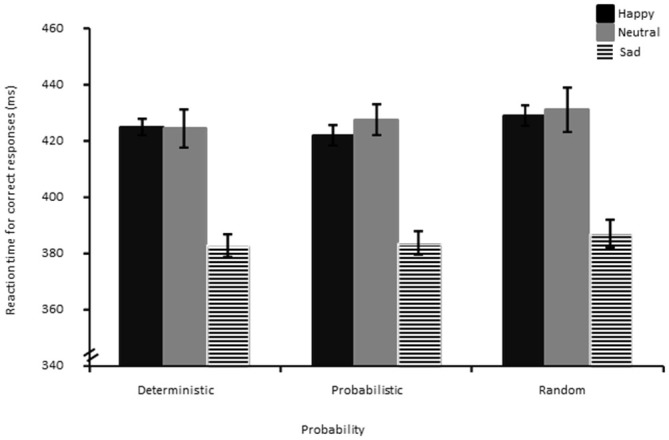
**Reaction times for correct responses decomposed as a function of bin, probability and group.** The error bar corresponds to one standard error of the mean.

Results showed a significant effect of Probability (*F*_(1.95,81.95)_ = 3.78, *p* < 0.05, η^2^ = 0.08). Follow-up *t*-tests showed that the random condition (*M* = 412.61, SD = 39.32) had marginally significantly longer RTs than the probabilistic (*M* = 408.62, SD = 35.66), (*t*_(44)_ = −1.85, *p* = 0.07). RTs for the random condition were significantly longer than for the deterministic condition (*M* = 406.78, SD = 35.22), (*t*_(44)_ = −3.05, *p* < 0.01). The deterministic and probabilistic conditions did not differ significantly from each other (*p* > 0.05). Unexpectedly, a significant main effect of Group was evidenced, (*F*_(2,42)_ = 10.82, *p* < 0.01, η^2^ = 0.34). Follow-up independent *t*-tests showed that the sad group (*M* = 382.81, SD = 34.20) had overall significantly faster RTs (see Figure 4) than the happy (*M* = 424.22, SD = 19.81), (*t*_(29)_ = 4.00, *p* < 0.01) and the neutral mood group (*M* = 426.68, SD = 32.35), (*t*_(29)_ = 3.64, *p* < 0.01).

### Learning Rate

**Figure 5:**
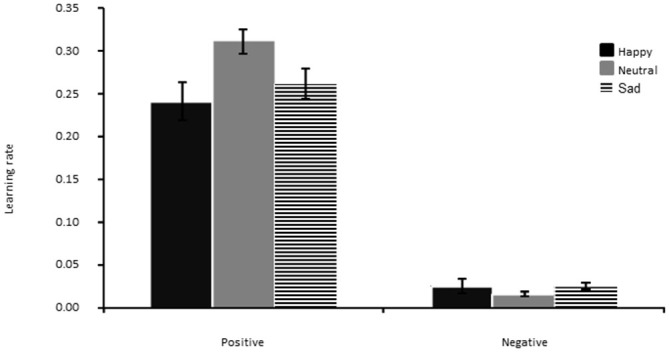
**Learning rate for positive (left panel) and negative (right panel) feedback, separately for the Happy, Neutral and Sad mood group.** The error bar corresponds to one standard error of the mean.

The main effect of feedback valence was significant (*F*_(1,42)_ = 172.78, *p* < 0.01, η^2^ = 0.80), showing that this parameter was overall larger for positive than negative feedback, as already found in our previous study (Bakic et al., 2014). Other effects remained all non-significant (all *p*^′^s > 0.05; see Figure 5).

### Exploration Parameter

**Figure 6:**
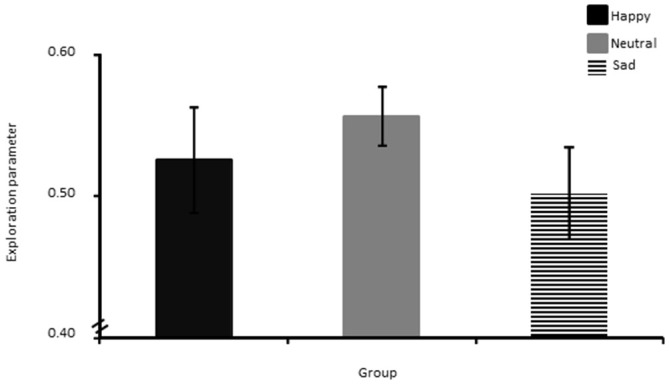
**Exploration parameter shown separately for the Happy, Neutral and Sad mood group.** The error bar corresponds to one standard error of the mean.

The one way ANOVA showed no significant group differences in exploration (Figure 6).

### Post-Experiment Ratings

The mixed-model ANOVA carried out on the clarity ratings showed a significant main effect of Probability (*F*_(1.70,71.61)_ = 708.28, *p* < 0.01 η^2^ = 0.94), showing that clarity increased monotonically as a function of increasing reward probability (Table 5). The analysis performed on the certainty ratings revealed a significant Phase*Probability interaction (*F*_(2,84)_ = 76.97, *p* < 0.01, η^2^ = 0.65), as well as significant main effects of Phase (*F*_(1,42)_ = 53.12, *p* < 0.01, η^2^ = 0.56) and of Probability (*F*_(2,84)_ = 228.89, *p* < 0.01, η^2^ = 0.85). This significant interaction was followed up by a paired *t*-tests to compare certainty across the two phases for each probability separately. The only significant difference was found in the deterministic condition, where certainty in the no feedback Phase (*M* = 82.93, SD = 5.66) was significantly higher than in the Phase with feedback (*M* = 61.49, SD = 11.48), (*t*_(44)_ = −15.32, *p* < 0.01).

**Table 5:** **Results of the Certainty and Clarity ratings**.

	Condition			*t*-test	
	Deterministic	Probabilistic	Random	Deterministic-Probabilistic	Probabilistic-Random
Certainty	82.64 (5.43)	73.40 (9.94)	36.18 (6.68)	7.47**	24.58**
Clarity	72.16 (7.76)	47.47 (11.44)	33.03 (12.07)	14.80**	7.08**

Means Means (+1 Standard Deviation) and results of the comparisons between conditions (based on t-tests, df = 44). *p < 0.05, **p < 0.01.

The analyses pertaining to subjective reports about the amount of positive vs. negative feedback received during the whole experimental session, as well as the like/dislike reactions to them revealed no significant group differences (all *p*’s > 0.05).

### Questionnaires

There were no significant group differences on BDI or RS-nl.

## Discussion

In this study, we sought to assess whether happy or sad mood could change RL, when compared to an active control condition or group with a neutral mood content. Even though no general consensus has emerged yet in the literature regarding effects of mood valence on learning (Gray, 2001; Nadler et al., 2010; van Steenbergen et al., 2010; Huntsinger, 2012), it is usually agreed that being in a state of increased emotionality (either positive or negative) alters motivational processes activated by cues signaling reward or punishment (Lang and Bradley, 2010), and hence learning by extension when this process is based on the direct exploitation of these incentives, like in the present case. More specifically, our primary goal was to assess if inducing happy mood could eventually lead to a gain in performance during RL, especially when externally-provided feedback information on task performance (hence cues signaling reward or punishment) were omitted and exploitation of learned reward probabilities was fostered. For this purpose, we adapted a previously validated probabilistic learning task (Eppinger et al., 2008; Unger et al., 2012; Bakic et al., 2014) and introduced a second phase during the experiment where feedback on task performance was removed and hence learning could no longer be based on these (external) incentives, i.e., negative or positive feedback regarding task performance. During the initial phase of the experiment where this feedback information was still available, we expected to replicate the results of our previous study (Bakic et al., 2014), where we found that inducing positive mood led to an increase in the learning rate. Based on this previous study as well as the evidence currently available in the literature, we formulated a specific prediction: if the valence of the mood plays an important role in RL (Bolte and Goschke, 2010; Chiew and Braver, 2014), then happy and sad participants should behave in opposite ways. More specifically, we expected that the learning rate would be larger in the happy compared to the neutral mood group, while it would be lower in the sad mood group compared to the control mood group. Moreover, by removing feedback as soon as learning was established, we hoped to exacerbate possible mood-related group differences in RL, bearing in mind that only standard accuracy and RT data could be extracted during this specific phase of the experiment (while computational modeling parameters could be estimated during the first phase of the experiment only, as in our previous study; see Bakic et al., 2014).

The results of this study confirm that guided imagery provides a valid method to induce and maintain specific mood states, characterized either by happiness or sadness. The happy mood group had a substantial increase in self-reported levels of happiness, comparable in size to the increase of sadness in the sad mood group.

Learning was clearly evidenced during the first phase of the experiment, equally strongly in the three mood groups however, challenging our assumption that mood valence (either positive or negative) could influence this process. Moreover, when considering two standard learning parameters extracted from a computational model (Jepma and Nieuwenhuis, 2011; Bakic et al., 2014), we still failed to reveal significant group differences, unlike what we found in our previous study where happy mood was associated with a larger learning rate (without concurrent change in exploration) compared to neutral mood. Strikingly, our results for the second phase of the experiment showed that participants (in all three groups) continued to learn in the absence of direct feedback information regarding task performance, suggesting that they unambiguously used or exploited abstract mental representations to comply with the task demands, as opposed to using or exploring externally provided cues signaling punishment or reward solely or primarily. However, neither happy nor sad mood did influence this learning process operating without exploration (second phase of the experiment).

Nieuwenhuis et al. (2005) previously discussed the importance of feedback delivery and content: when it is delivered on a trial level, subjects tend to rely on external information more than on the internal monitoring system, and this may lead to reduced uncertainty levels as there is always an external check of the prediction. When the feedback is removed, it is expected that internal monitoring processes and the knowledge of the associations will be even more activated. Our current results add indirect support to this claim as we saw that removing feedback content did not lead to a cost, but learning still progressed as if a boost of exploitation was triggered by this manipulation.

**Figure 7:**
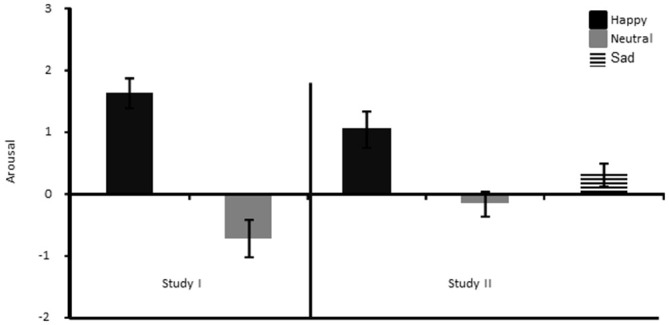
**Arousal ratings shown separately for the Happy and the Neutral mood group of Bakic et al. (2014) (left panel), and the three different mood groups of the current study (right panel)**.

The failure to replicate our previous findings for the learning rate parameter during the first phase of the experiment (see Bakic et al., 2014) is puzzling at first sight, given that aside from the inclusion of a sad mood group in the current study, the experimental procedure was kept identical between these two studies for this phase. However, a closer look at the subjective ratings in these two studies might give us some hints on some of the reasons underlying this apparent discrepancy. When comparing mood changes directly between the two studies (i.e., the present one and Bakic et al., 2014), we found that the MIP in our previous study led to a large and significant difference between the two mood groups (happy and neutral) not only in valence, but also in arousal (*t*_(30)_ = 3.10, *p* < 0.01), while this was not the case in the current study (*p* = 0.10; see Figure 7). Hence, in our previous study (Bakic et al., 2014), participants in the happy mood group were not only more happy than in the neutral mood group, but also more aroused by the MIP; an effect that was not found in the current study. Moreover, in our previous study, we found that the increase in happiness following the MIP (relative to the pre-MIP baseline measurement) correlated strongly with the increase in arousal (*r* = 0.63, *p* < 0.01), and importantly with both the positive (*r* = 0.44, *p* < 0.01) and the negative learning rate (*r* = 0.51, *p* < 0.01) as well. Hence, in our previous study (Bakic et al., 2014), the higher learning rate found in the positive than in the neutral mood group was likely explained by changes occurring both in valence and arousal as a function of the MIP. By comparison, no similar correlation was evidenced in the current study. Likewise, we also failed to find evidence in this study for significant group differences regarding the perceived amount and like/dislike reactions to the feedback given during the RL task, while we did so in our previous study (see Bakic et al., 2014), suggesting that the elected MIP had probably a different and stronger impact (in terms of emotional changes brought about) in the positive mood group in our previous compared to the current study. Accordingly, it is tempting to conclude that our failure to replicate our previous findings for the learning rate (which was increased in the happy compared to the neutral mood group; see Bakic et al., 2014) in the present study might be imputed to the failure to elicit a reliable increase in levels of arousal in the happy compared to the neutral or sad mood group with our MIP. More generally, we believe this systematic comparison between our two studies is valuable because it confirms that arousal is probably an important dimension to consider (besides valence *per se*) in order to better understand modulatory effects on RL as a function of positive mood, as we previously observed (Bakic et al., 2014) but failed to replicate here. However, it should be added that in the domain of creative thinking, earlier studies already showed that (positive) valence, rather than arousal *per se*, was accompanied by a gain in performance (Isen et al., 1987), suggesting that during the encounter of positive mood or affect, valence and arousal could very well have different effects depending on the specific context and task demands. As a matter of fact, arousal has often been conceived as an important determinant of learning (and more specifically the exploration-exploitation trade-off) in the past, providing salience information to the organism. For example, according to the adaptive gain theory of Aston-Jones and Cohen (2005), exploration and exploitation are two decision strategies that depend directly on tonic and phasic changes in arousal, which is modulated by salience estimation generated in prefrontal areas. Jepma and Nieuwenhuis (2011) previously used this specific framework using a “four-armed bandit” task in healthy adult participants (without any mood induction) and showed that changes in the pupil diameter (a putative index of locus coeruleus activity) correlated with transitions from exploration to exploitation. A recent study corroborated the assumption that arousal-related processes (captured by the pupil size) indeed contributed to shape learning in a volatile setting (Browning et al., 2015). Moreover, Fröber and Dreisbach (2012) recently confirmed the importance of arousal during the experience of positive affect to account for modulatory effects of the current affective state on proactive control mechanisms. Speculatively, it may therefore be the case that our MIP in the present study failed to increase arousal substantially in the happy mood group (unlike what we found in Bakic et al., 2014), which in turn did not change the exploration-exploitation trade-off and/or the learning rate in this group. Alternatively, arousal (resulting from the MIP we used here and in our previous study) could also foster probabilistic learning, when elicited to a sufficient degree, by influencing specific (short-term) memory processes needed to resolve the task, given that arousal usually heightens memory (Mather and Carstensen, 2005; Clewett and Mather, 2014). At any rate, future studies are needed to assess whether (positive) mood valence could create changes in RL if and only if this specific mood state is accompanied by variations along the arousal dimension too.

Several limitations of our study warrant comment. First, if arousal plays an important role in mediating effects of mood on RL, then it is likely that a MIP tailored to increase arousal selectively (rather than valence) might provide a more promising avenue to evidence effects of mood on the exploration-exploitation trade-off during RL (Knutson et al., 2014). Here by contrast, we created three groups differing primarily regarding the valence of the mood induced (happy, sad or neutral), which may eventually have blurred rather than cleared some of the group differences during RL. The choice of a (low-arousing) sad mood as comparison for the happy mood group was motivated by many earlier studies and models in the literature arguing that sadness can be conceived as the opposite of happiness, as well as a good proxy of the anhedonic component in depression, for which there is already good evidence for modulatory effects on learning behavior, especially when it is based on either reward or punishment incentives/cues (Taylor Tavares et al., 2008; Chase et al., 2010; Padrão et al., 2013; Liu et al., 2014; Pizzagalli, 2014). However, in our study, despite the successful experience of sad mood, we note that participants from this group did not perform worse during the probabilistic learning task (for none of the two phases) than the two other mood groups, casting doubt in turn on the notion that effects of sad mood on learning and cognition can simply be opposed to effects associated with happy mood.

Second, even though happy or sad mood did not alter learning at the behavioral (or computational modeling) level, we cannot rule out the possibility that these mood states could influence specific electrophysiological markers of RL, including the ERN and FRN components. Noteworthy, in our previous study (Bakic et al., 2014), we found that happy compared to neutral mood increased the ERN component in the deterministic condition selectively. Accordingly, it could be valuable in future studies to add EEG correlates of RL and performance monitoring (Koban and Pourtois, 2014) in order to assess whether mood could alter early stages of error monitoring (in the absence of obvious changes at the behavioral level) or not, especially when feedback information on task performance is removed and learning has therefore to operate primarily based on the direct exploitation of known S-R associations (carrying a high reward probability).

Finally, the probabilistic learning task used here (relying on a simple speeded two-alternatives forced choice task; see Eppinger et al., 2008) may not be sensitive enough to capture subtle changes in learning related to labile mood states, such as elicited after the MIP (based on guided imagery) used in this study. Perhaps mood does change choice behavior, but not decision making *per se*, a hypothesis that would require the use of other experimental paradigms than the one used here, and where not only the amount but also the type of learning strategy at stake could be probed (see Frank et al., 2015). Presumably, the specific structure of the task used here, as well as the specific task requirements, may have weakened the expression of mood-related changes during RL. Likewise, reversal learning paradigms (see Chase et al., 2011) or more complex and volatile learning environments based on the use of more than two-alternatives forced choice task (see Jepma and Nieuwenhuis, 2011; Browning et al., 2015) could perhaps help to reveal clearer and stronger modulatory effects of either positive or negative mood on RL.

## Funding

Concerted Research Action Grant from Ghent University, BOF10/GOA/014. Belgian Science Policy, Interuniversity Attraction Poles program, P7/11.

## Conflict of Interest Statement

The authors declare that the research was conducted in the absence of any commercial or financial relationships that could be construed as a potential conflict of interest.

## References

[B1] Ashby F. G. Isen A. M. Turken U. (1999). A neuropsychological theory of positive affect and its influence on cognition. Psychol. Rev. 106, 529–550. 10.1037/0033-295x.106.3.529 10467897

[B2] Aston-Jones G. Cohen J. D. (2005). An integrative theory of locus coeruleus-norepinephrine function: adaptive gain and optimal performance. Annu. Rev. Neurosci. 28, 403–450. 10.1146/annurev.neuro.28.061604.135709 16022602

[B3] Bakic J. Jepma M. De Raedt R. Pourtois G. (2014). Effects of positive mood on probabilistic learning: behavioral and electrophysiological correlates. Biol. Psychol. 103, 223–232. 10.1016/j.biopsycho.2014.09.012 25265572

[B4] Beck A. T. Steer R. A. Ball R. Ranieri W. (1996). Comparison of beck depression inventories -IA and -II in psychiatric outpatients. J. Pers. Assess. 67, 588–597. 10.1207/s15327752jpa6703_13 8991972

[B5] Behrens T. E. J. Woolrich M. W. Walton M. E. Rushworth M. F. S. (2007). Learning the value of information in an uncertain world. Nat. Neurosci. 10, 1214–1221. 10.1038/nn1954 17676057

[B6] Bolte A. Goschke T. (2010). “Thinking and emotion: affective modulatoin of cognitive processing modes,” in Towards a Theory of Thinking, eds Glatzeder B. Goel V. Müller A. (Berlin: Springer Berlin Heidelberg), 261–277.

[B7] Braem S. Verguts T. Roggeman C. Notebaert W. (2012). Reward modulates adaptations to conflict. Cognition 125, 324–332. 10.1016/j.cognition.2012.07.015 22892279

[B8] Browning M. Behrens T. E. Jocham G. O’Reilly J. X. Bishop S. J. (2015). Anxious individuals have difficulty learning the causal statistics of aversive environments. Nat. Neurosci. 18, 590–596. 10.1038/nn.3961 25730669 PMC4644067

[B9] Cavanagh J. F. Bismark A. J. Frank M. J. Allen J. J. B. (2011). Larger error signals in major depression are associated with better avoidance learning. Front. Psychol. 2:331. 10.3389/fpsyg.2011.00331 22084638 PMC3210982

[B10] Chase H. W. Frank M. J. Michael A. Bullmore E. T. Sahakian B. J. Robbins T. W. (2010). Approach and avoidance learning in patients with major depression and healthy controls: relations to anhedonia. Psychol. Med. 40, 433–440. 10.1017/s0033291709990468 19607754

[B11] Chase H. W. Swainson R. Durham L. Benham L. Cools R. (2011). Feedback-related negativity codes prediction error but not behavioral adjustment during probabilistic reversal learning. J. Cogn. Neurosci. 23, 936–946. 10.1162/jocn.2010.21456 20146610

[B12] Chiew K. S. Braver T. S. (2014). Dissociable influences of reward motivation and positive emotion on cognitive control. Cogn. Affect. Behav. Neurosci. 14, 509–529. 10.3758/s13415-014-0280-0 24733296 PMC4072919

[B13] Clewett D. V. Mather M. (2014). Not all that glittered is gold: neural mechanisms that determine when reward will enhance or impair memory. Front. Neurosci. 8:194 . 10.3389/fnins.2014.00194 25076871 PMC4099934

[B14] Cohen J. D. McClure S. M. Yu A. J. (2007). Should I stay or should I go? How the human brain manages the trade-off between exploitation and exploration. Philos. Trans. R. Soc. Lond. B Biol. Sci. 362, 933–942. 10.1098/rstb.2007.2098 17395573 PMC2430007

[B15] Dreisbach G. (2006). How positive affect modulates cognitive control: the costs and benefits of reduced maintenance capability. Brain Cogn. 60, 11–19. 10.1016/j.bandc.2005.08.003 16216400

[B16] Eppinger B. Kray J. Mock B. Mecklinger A. (2008). Better or worse than expected? Aging, learning and the ERN. Neuropsychologia 46, 521–539. 10.1016/j.neuropsychologia.2007.09.001 17936313

[B17] Frank M. J. Gagne C. Nyhus E. Masters S. Wiecki T. V. Cavanagh J. F.. (2015). fMRI and EEG predictors of dynamic decision parameters during human reinforcement learning. J. Neurosci. 35, 485–494. 10.1523/JNEUROSCI.2036-14.2015 25589744 PMC4293405

[B18] Fredrickson B. L. (2004). The broaden-and-build theory of positive emotions. Philos. Trans. R. Soc. Lond. B Biol. Sci. 359, 1367–1378. 10.1098/rstb.2004.1512 15347528 PMC1693418

[B20] Fröber K. Dreisbach G. (2012). How positive affect modulates proactive control: reduced usage of informative cues under positive affect with low arousal. Front. Psychol. 3:265. 10.3389/fpsyg.2012.00265 22866047 PMC3406411

[B19] Fröber K. Dreisbach G. (2014). The differential influences of positive affect, random reward and performance-contingent reward on cognitive control. Cogn. Affect. Behav. Neurosci. 14, 530–547. 10.3758/s13415-014-0259-x 24659000

[B21] Gray J. R. (2001). Emotional modulation of cognitive control: approach-withdrawal states double-dissociate spatial from verbal two-back task performance. J. Exp. Psychol. Gen. 130, 436–452. 10.1037/0096-3445.130.3.436 11561919

[B23] Holmes E. A. Coughtrey A. E. Connor A. (2008). Looking at or through rose-tinted glasses? Imagery perspective and positive mood. Emotion 8, 875–879. 10.1037/a0013617 19102599

[B24] Holmes E. A. Mathews A. Dalgleish T. Mackintosh B. (2006). Positive interpretation training: effects of mental imagery versus verbal training on positive mood. Behav. Ther. 37, 237–247. 10.1016/j.beth.2006.02.002 16942975

[B22] Holmes E. A. Mathews A. (2010). Mental imagery in emotion and emotional disorders. Clin. Psychol. Rev. 30, 349–362. 10.1016/j.cpr.2010.01.001 20116915

[B25] Hunsinger M. Isbell L. M. Clore G. L. (2012). Sometimes happy people focus on the trees and sad people focus on the forest: context-dependent effects of mood in impression formation. Pers. Soc. Psychol. Bull. 38, 220–232. 10.1177/0146167211424166 21957087 PMC4116487

[B26] Huntsinger J. R. (2012). Does positive affect broaden and negative affect narrow attentional scope? A new answer to an old question. J. Exp. Psychol. Gen. 141, 595–600. 10.1037/a0027709 22409665

[B27] Isen A. M. (1984). “Toward understnding the role of affect in cognition,” in Handbook of Social Cognition, eds Wyer R. Srull T. (Hillsdale, NJ: Erlbaum), 174–236.

[B28] Isen A. M. (1993). “Positive affect and decision making,” in Handbook of Emotion, eds Lewis M. Haviland J. (New York: Guilford Press), 261–277.

[B29] Isen A. M. Daubman K. A. Nowicki G. P. (1987). Positive affect facilitates creative problem solving. J. Pers. Soc. Psychol. 52, 1122–1131. 10.1037/0022-3514.52.6.1122 3598858

[B30] Isen A. M. Johnson M. M. Mertz E. Robinson G. F. (1985). The influence of positive affect on the unusualness of word associations. J. Pers. Soc. Psychol. 48, 1413–1426. 10.1037/0022-3514.48.6.1413 4020605

[B31] Jepma M. Nieuwenhuis S. (2011). Pupil diameter predicts changes in the exploration-exploitation trade-off: evidence for the adaptive gain theory. J. Cogn. Neurosci. 23, 1587–1596. 10.1162/jocn.2010.21548 20666595

[B32] Knutson B. Katovich K. Suri G. (2014). Inferring affect from fMRI data. Trends Cogn. Sci. 18, 422–428. 10.1016/j.tics.2014.04.006 24835467

[B33] Koban L. Pourtois G. (2014). Brain systems underlying the affective and social monitoring of actions: an integrative review. Neurosci. Biobehav. Rev. 46, 71–84. 10.1016/j.neubiorev.2014.02.014 24681006

[B34] Lang P. J. Bradley M. M. (2010). Emotion and the motivational brain. Biol. Psychol. 84, 437–450. 10.1016/j.biopsycho.2009.10.007 19879918 PMC3612949

[B35] Liu W.-H. Wang L.-Z. Shang H.-R. Shen Y. Li Z. Cheung E. F. C.. (2014). The influence of anhedonia on feedback negativity in major depressive disorder. Neuropsychologia 53, 213–220. 10.1016/j.neuropsychologia.2013.11.023 24316199

[B36] Martin M. (1990). On the induction of mood. Clin. Psychol. Rev. 10, 669–697. 10.1016/0272-7358(90)90075-L

[B37] Mather M. Carstensen L. L. (2005). Aging and motivated cognition: the positivity effect in attention and memory. Trends Cogn. Sci. 9, 496–502. 10.1016/j.tics.2005.08.005 16154382

[B38] Nadler R. T. Rabi R. Minda J. P. (2010). Better mood and better performance. Learning rule-described categories is enhanced by positive mood. Psychol. Sci. 21, 1770–1776. 10.1177/0956797610387441 20974709

[B39] Nieuwenhuis S. Nielen M. M. Mol N. Hajcak G. Veltman D. J. (2005). Performance monitoring in obsessive-compulsive disorder. Psychiatry Res. 134, 111–122. 10.1016/j.psychres.2005.02.005 15840412

[B40] Padrão G. Mallorquí A. Cucurell D. Marco-Pallares J. Rodriguez-Fornells A. (2013). Neurophysiological differences in reward processing in anhedonics. Cogn. Affect. Behav. Neurosci. 13, 102–115. 10.3758/s13415-012-0119-5 22968926

[B41] Pizzagalli D. A. (2014). Depression, stress and anhedonia: toward a synthesis and integrated model. Annu. Rev. Clin. Psychol 10, 393–423. 10.1146/annurev-clinpsy-050212-185606 24471371 PMC3972338

[B42] Portzky M. Wagnild G. De Bacquer D. Audenaert K. (2010). Psychometric evaluation of the dutch resilience scale RS-nl on 3265 healthy participants: a confirmation of the association between age and resilience found with the swedish version. Scand. J. Caring Sci. 24(Suppl. 1), 86–92. 10.1111/j.1471-6712.2010.00841.x 21070312

[B43] Rossion B. Pourtois G. (2004). Revisiting snodgrass and vanderwart’s object pictorial set: the role of surface detail in basic-level object recognition. Perception 33, 217–236. 10.1068/p5117 15109163

[B44] Sutton R. Barto A. G. (1998). Reinforcement Learning: An Introduction. Cambridge, MA: MIT Press.

[B45] Taylor Tavares J. V. Clark L. Furey M. L. Williams G. B. Sahakian B. J. Drevets W. C. (2008). Neural basis of abnormal response to negative feedback in unmedicated mood disorders. Neuroimage 42, 1118–1126. 10.1016/j.neuroimage.2008.05.049 18586109 PMC2745889

[B46] Unger K. Kray J. Mecklinger A. (2012). Worse than feared? Failure induction modulates the electrophysiological signature of error monitoring during subsequent learning. Cogn. Affect. Behav. Neurosci. 12, 34–51. 10.3758/s13415-011-0061-y 21960022

[B47] van Steenbergen H. Band G. P. Hommel B. (2010). In the mood for adaptation: how affect regulates conflict-driven control. Psychol. Sci. 21, 1629–1634. 10.1177/0956797610385951 20943936

[B48] Vanlessen N. De Raedt R. Mueller S. C. Rossi V. Pourtois G. (2015). Happy and less inhibited? Effects of positive mood on inhibitory control during an antisaccade task revealed using topographic evoked potential mapping. Biol. Psychol. 110, 190–200. 10.1016/j.biopsycho.2015.07.004 26196900

[B49] Vanlessen N. Rossi V. De Raedt R. Pourtois G. (2013). Positive emotion broadens attention focus through decreased position-specific spatial encoding in early visual cortex: evidence from ERPs. Cogn. Affect. Behav. Neurosci. 13, 60–79. 10.3758/s13415-012-0130-x 23090718

[B50] Vanlessen N. Rossi V. De Raedt R. Pourtois G. (2014). Feeling happy enhances early spatial encoding of peripheral information automatically: electrophysiological time-course and neural sources. Cogn. Affect. Behav. Neurosci. 14, 951–969. 10.3758/s13415-014-0262-2 24570275

[B51] Watkins E. R. Moberly N. J. (2009). Concreteness training reduces dysphoria: a pilot proof-of-principle study. Behav. Res. Ther. 47, 48–53. 10.1016/j.brat.2008.10.014 19036353 PMC2807031

[B52] Westermann R. Stahl G. Hesse F. W. (1996). Relative effectiveness and validity of mood induction procedures: a meta-analysis. Eur. J. Soc. Psychol. 26, 557–580. 10.1002/(sici)1099-0992(199607)26:4<557::aid-ejsp769>3.0.co;2-4

[B53] Zwosta K. Hommel B. Goschke T. Fischer R. (2013). Mood states determine the degree of task shielding in dual-task performance. Cogn. Emot. 27, 1142–1152. 10.1080/02699931.2013.772047 23438389

